# Dual Specificity Phosphatases: From Molecular Mechanisms to Biological Function

**DOI:** 10.3390/ijms20184372

**Published:** 2019-09-06

**Authors:** Rafael Pulido, Roland Lang

**Affiliations:** 1 Biomarkers in Cancer Unit, Biocruces Bizkaia Health Research Institute, 48903 Barakaldo, Spain; 2 IKERBASQUE, Basque Foundation for Science, 48011 Bilbao, Spain; 3 Institute of Clinical Microbiology, Immunology and Hygiene, Universitätsklinikum Erlangen, Friedrich-Alexander-Universität Erlangen-Nürnberg, 91054 Erlangen, Germany

Dual specificity phosphatases (DUSPs) constitute a heterogeneous group of enzymes, relevant in human disease, which belong to the class I Cys-based group of protein tyrosine phosphatase (PTP) gene superfamily [1,2,3,4]. DUSPs possess the capability to dephosphorylate Ser/Thr and Tyr residues from proteins as well as to remove phosphates from other non-proteinaceous substrates, including signaling lipids [5]. Catalytically inactive pseudophosphatase DUSPs also exist which regulate phosphorylation-related cell signaling [6]. DUSPs include, among others, mitogen-activated protein kinase (MAPK) phosphatases (MKPs) and small-size atypical DUSPs. These proteins are non-transmembrane enzymes displaying variable substrate specificity and harboring a single PTP catalytic domain with a HCXXGXXR consensus catalytic motif [7,8]. MKPs are enzymes specialized in regulating the catalytic activity and subcellular location of MAPKs, whereas the functions of small-size atypical DUSPs are more diversified. DUSPs have emerged as key players in the regulation of cell growth, differentiation, stress responses and apoptosis. In physiology, DUSPs regulate essential processes, including immunity, neurobiology and metabolic homeostasis, and have been involved in tumorigenesis, pathological inflammation and metabolic disorders [9,10,11]. Accordingly, alterations in the expression or function of MKPs and small-size atypical DUSPs have important consequences in human disease, making these enzymes potential biological markers and therapeutic targets. Although major biochemical, structural, functional and physiological properties of many DUSPs are currently known, their developmental stage- and tissue-specific involvement in different human pathologies is only starting to be disclosed. This Special Issue provides original research and review articles focused on the involvement of specific MKPs and small-size atypical DUSPs in human disease, including relevant information on the use of different biological models to study the regulation and physiological functions of DUSP enzymes. 

MKPs are also present in fungi, where they are major regulators of the different fungal MAPK adaptive pathways [12]. An update of the repertoire of MKPs in pathogenic and non-pathogenic fungi is presented, together with their functional effects on MAPK signaling in fungi and insights into their regulatory mechanisms of expression and function. The budding yeast *Saccharomyces cerevisiae* (with two active MKPs, Msg5 and Sdp1) and the fission yeast *Schyzosaccharomyces pombe* (with one active MKP, Pmp1) emerge as suitable models to study MKP regulation and function, with potential translation to other eukaryotic organisms [13].

The roles of MKPs and small-size atypical DUSPs in MAPK-dependent and -independent immune cell response have been reviewed [14,15]. In the context of airway epithelial signaling during viral infection in inflammatory airway processes, such as asthma and chronic obstructive pulmonary disease, several MKPs, including DUSP1, DUSP4 and DUSP10, arise as major negative regulators of inflammation due to their inhibitory effects on the major pro-inflammatory Jun N-terminal kinase (JNK), p38, and Extracellular signal-regulated kinase (ERK) MAPKs in the airway epithelium. Up-regulation or regulated activation of these MKPs constitute potential anti-inflammatory therapeutic approaches for inflammatory airway diseases [15]. Although the more frequently documented effects of MKPs in immunity involve direct dephosphorylation of MAPKs, the existence of non-MAPK MKP and small-size atypical DUSP substrates is also disclosed. The complexity and redundancy in DUSP signaling during immune cell response make dedicated studies and testing necessary, in appropriate biological models, for both the inhibition and activation of DUSPs as suitable therapeutic strategies for immune diseases [14]. In this regard, a comprehensive in silico study is presented analyzing the expression and functional interaction between DUSPs and protein kinases in hematopoietic cells, which unveils the interplay between DUSPs and novel non-MAPK protein kinases, including receptor tyrosine kinases (IGFR1, VEGF, FGF), AURKA, and LRRK2, among others [16]. In addition, the control of DUSPs protein stability by ubiquitination and phosphorylation has been reviewed as a major regulatory mechanism affecting most MKPs, whereas methylation-induced ubiquitination of DUSP14 is disclosed as a specific mechanism to activate the catalysis of this small-size atypical DUSP [17].

In a more specific context, the complex expression and regulation patterns of DUSP10, and its diverse functional roles in inflammation, immunity, and cancer, which could go beyond direct MAPK dephosphorylation, has been separately reviewed [18]. Li et al. presented their findings on the effects on gene expression and lipid metabolism of *Escherichia coli*-triggered sepsis, using a *Dusp1*^−/−^ mouse model [19], whereas Neamatallah et al. described their findings on the macrophage gene expression profiles on *Dusp4*^−/−^ mice [20]. Circulating tumor cells play a major role in tumor dissemination and metastasis, and Wu et al. reported the enrichment in nuclear localized DUSP6 in circulating tumor cells from triple negative breast cancers, as well as in brain metastases, suggesting a specific role for nuclear DUSP6 in cancer spreading [21]. Finally, Cao et al. provided new insights into the functions of the catalytically inactive MK-STYX pseudophosphatase as an indirect regulator of post-translational modifications from proteins regulating microtubule dynamics, including histone deacetylase isoform 6 and tubulin [22]. 

Neuroblastoma constitutes the most commonly diagnosed extracranial solid tumor in infants. The current knowledge on the involvement of MKPs and small-size atypical DUSPs in neuroblastoma cell growth and differentiation, in the context of Trks, STATs and ALK/RAS/MAPK signaling, has been covered. Highlights are made on the potential role of ERK1/2-specific DUSP5 and DUSP6 as neuroblastoma biomarkers, as well as on the potential of inhibition of other MKPs, such as DUSP1, DUSP8, DUSP10, or DUSP16 for therapy of neuroblastoma [23]. Further knowledge into the roles of MKPs in neuronal differentiation and nervous system development has also been reviewed by Perez-Sen et al., with emphasis in MKP-mediated neuroprotection upon genotoxic and ischemic neuron injury. Cell-specific signaling through neurotrophins, cannabinoids and nucleotides may have neuroprotective effects by up-regulation of specific MKPs. In addition, the dual anti- or pro-oncogenic role of DUSP1 and DUSP6 in glioblastoma, the most aggressive type of brain tumor, is discussed [24].

In summary, this Special Issue provides an updated overview on the complexity of DUSP biology at the physiological level, which is a prerequisite for the validation of DUSPs as useful biomarkers or drug-targetable proteins in human disease treatment. The direct functional relation between many of the DUSPs and MAPKs provides a high therapeutic potential for DUSP proteins, which is evident in the MKP DUSP subfamily. However, DUSPs redundancy, multiplicity in MAPK substrate specificity, and time-course and subcellular localization functional constraints, make it difficult to link unequivocally DUSPs expression and function with pathological manifestations. Well-defined biological models with precisely manipulated DUSP protein expression and function, as well as accurate molecular definition of functional DUSPs partners, with special emphasis on orphan small-size atypical DUSPs, will help in the future progress of the field.

We thank all the colleagues that contributed with their work and expertise to this Special Issue, and we hope that its content is of interest for clinicians and researchers aiming to explore and understand the role of DUSPs in human disease, as well as the potential benefits of their therapeutic manipulation.
